# Electrically Conductive Functional Polymers and Application Progress in Lithium Batteries

**DOI:** 10.3390/polym17060778

**Published:** 2025-03-14

**Authors:** Zhe Huang, Mengting Lyu, Nan Meng, Jinxin Cao, Chenyu Xiong, Fang Lian

**Affiliations:** School of Materials Science and Engineering, University of Science and Technology, Beijing 100083, China; zhehuang0722@163.com (Z.H.); 15052150017@163.com (M.L.); mengnan@ustb.edu.cn (N.M.); cjxys1998@163.com (J.C.); chenyuxiong_x@163.com (C.X.)

**Keywords:** lithium batteries, polymers, electrically conductive, multifunctional, redox activity, flexibility

## Abstract

Electrically conductive functional polymers (ECFPs) have attracted much attention not only for their electron conductivity but also for their versatile properties, including redox activity, flexibility, and designability. These attributes are expected to enhance the energy density and mechanical compatibility of lithium batteries while mitigating the safety risks associated with such batteries. Furthermore, ECFPs are key candidates as active materials, current collectors, coatings, binders, and additives in energy storage and conversion systems, especially for the development of flexible batteries, dry electrodes, and solid-state batteries. However, their low electron conductivity, poor environmental stability, instability of dopants, and high costs limit their usage in production and large-scale applications. In this review, the two major electrically conductive functional polymer species with conjugated and radical structures are focused on to reveal their conductivity mechanisms. Moreover, the current strategies for improving the performance of these polymers are summarized, which include molecular design to optimize conjugated structures for enhanced conductivity, the addition of hydrophobic groups or protective coatings to improve environmental resistance, a side-chain design that is self-doping to introduce high-stability dopants, and the development of multifunctional systems through compositing with two-dimensional carbon-based materials. Additionally, green processes and renewable resource applications are also introduced with the aim of creating cost-effective and sustainable preparation technologies. The advancement of ECFPs in structural and performance engineering and optimization strategies will facilitate their potentially expansive applications in energy storage and conversion devices.

## 1. Introduction

Polymers were thought of as insulators until Alan MacDiarmid et al. found conductive polyacetylene in 1977 [1]. Subsequently, various polymers such as polyaniline and polypyrrole were discovered to have electronic conductivity [2,3]. Electrically conductive functional polymers (abbreviated as ECFPs) refer to specialized polymer materials that not only possess the pros of conventional polymers but also the ability to conduct electricity. Importantly, regarding their applications, ECFPs also exhibit inherent flexibility, easy processing characteristics, and certain mechanical strength [4,5]. Therefore, they are usually employed to construct the elastic interface for lithium batteries with a higher energy density and degree of safety, especially for flexible energy storage devices. The more important pros of ECFPs are their degradability, metal-free nature, and environmentally friendly usage. The degradability of ECFPs means that they are able to decompose naturally after completing their function, reducing environmental damage; the absence of metallic components reduces the long-term pollution of the environment compared to many traditional materials that rely on metal catalysts or additives; and the production process of ECFPs is generally greener, consuming less energy, emitting fewer emissions, and exhibiting environmental friendliness throughout their life cycle [6,7].

ECFPs are important in diverse energy and conversion systems, leveraging their redox properties. They find applications in lithium-ion batteries, lithium–sulfur batteries, sodium metal batteries, supercapacitors, and photovoltaic electrodes. Moreover, ECFPs are also utilized in the photovoltaic industry for imaging and organic solar cells, in the medical sector as magnetic resonance contrast agents and antioxidants, and in chemistry, where nitrogen–oxygen radical polymers are commonly employed as catalytic oxidants [8,9].

At present, the common preparation methods of ECFPs mainly include chemical oxidative polymerization [10], in situ polymerization [11], electrochemical polymerization methods [12], and copolymerization or composite strategies [13]. ECFPs exhibit a larger specific surface area compared to traditional carbon-based conductive agents, such as carbon black, thereby establishing extensive and continuous electron transport pathways in lithium batteries, both point-to-point and point-to-face. Consequently, ECFPs can form a stable layer at the interface between battery components, enhancing interface compatibility, reducing adverse reactions, decreasing interfacial resistance, and improving the safety and stability of the battery [14]. Moreover, ECFPs are modified to possess a dual-conductive capability and construct continuous electron and ion conduction networks in thick electrodes for high-energy-density batteries [15,16]. Additionally, some ECFPs substitute a portion of the binder, realizing the functional integration of battery materials and enhancing the mechanical strength of the electrodes. The inherent flexibility of polymers offers greater processing versatility, making them suitable for solvent-free electrode processes for the fabrication of flexible electrodes or electrolyte membranes [17,18,19]. Notably, their superior mechanical properties and high toughness enable them to effectively accommodate volume changes in electrode materials during charge and discharge cycles, mitigate stress impacts, and improve the cycling stability and lifespan of batteries.

**Figure 1:**
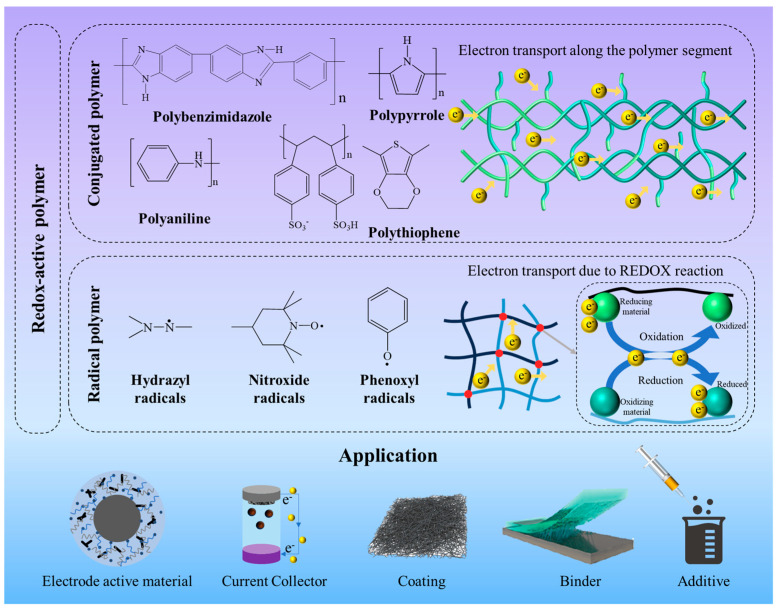
Typical electrically conducting polymers with conjugated and radical structures, their corresponding conduction mechanisms, and their applications in lithium batteries.

Polymers are generally classified into two main categories: redox-active and non-redox-active. Redox-active polymers are widely used in lithium batteries because of their optimal molecular structure, great stability, good chemical and physical properties, and excellent electrochemical storage ability. According to their molecular structure, redox-active polymers can be further divided into conjugate and radical systems [20]. As shown in Figure 1, typical conductive functional polymers with a conjugated structure, including polyaniline (PANI), poly(3,4-ethylenedioxythiophene) (PEDOT), polybenzimidazole (PBI), and polypyrrole (PPy), exhibit electrical characteristics comparable to those of inorganic materials like semiconductors and metals [21]. The electronic conduction mechanism of conjugated polymers mainly depends on their molecular structural characteristics and electronic properties. These polymers often have alternating single- and double-bond structures that allow their π electrons to move freely along the polymer chain, thereby facilitating electron conductivity. Due to their low density, excellent processability, and high mechanical flexibility, they can be shaped into various forms, such as films and fibers, which are crucial for advancements in modern electronic and optoelectronic materials science and technology. Meanwhile, it is worth noting that some radical polymers have been reported with good intrinsic conductivity [22]. The electron transfer in radical polymers mainly happens through the redox reactions of free radical groups, involving electron exchanges with either intermolecular or intramolecular bonds. In some free radical polymers, the presence of free radicals facilitates the formation of π-electron systems, which provide efficient pathways for electron migration. Although the π-π interactions in these polymers are generally weak, it is still possible to support electron migration under specific conditions [23,24,25]. In particular, radical polymers, characterized by a high density of redox-active sites, exhibit significant potential as electrode-active materials for energy storage and conversion devices [26]. For instance, poly(4-glycidyloxy-2,2,6,6-tetramethylpiperidine-1-oxyl) radical films with internal active sites for electron conduction are ultimately applied as anode materials in batteries [27]. Moreover, nitrogen–oxygen radicals are commonly used as catalytic oxidants in organic synthesis, catalysts in metal-based batteries, and active materials in redox flow batteries.

ECFPs possess conductivity, flexibility, and lightweight characteristics, which have garnered widespread attention. This review introduces and compares major conjugated polymers and radical polymer systems with their respective conduction mechanisms. In particular, the latest research advancements such as electrode active materials, binders, conductive agents, separator coatings, etc., in lithium batteries are reviewed in detail. Moreover, the current strategies employed to enhance electron conductivity and integrated performance are summarized, aiming to offer novel perspectives on the development and utilization of ECFPs in energy storage and conversion systems.

## 2. Conjugated Polymers

Conjugated polymers achieve conductive function from their long molecular chains with largely delocalized and polarized π-conjugated structures, which are composed of conjugated double bonds. Strategic molecular engineering of these π-conjugated backbones, particularly through π-orbital extension, significantly enhances charge transport capabilities [28,29]. In lithium battery systems, these polymers serve three principal functions: (i) As electrode active materials, their superior electrical conductivity facilitates efficient charge transfer, thereby improving electrochemical utilization kinetics and boosting both specific capacity and rate performance; (ii) As conductive additives, they enable precise modulation of electrode conductivity; (iii) When employed as polymeric binders, they enhance electrode mechanical integrity while maintaining flexibility, ultimately extending their life cycle. The subsequent sections systematically elaborate on representative conjugated polymer systems and their optimization methodologies.

### 2.1. PANI

MacDiarmid made the discovery of PANI in the 1980s, and it is well known for its remarkable electrochemical and redox properties, environmental stability, and high conductivity [30,31]. While pure PANI demonstrates insulating behavior, its conductivity enhancement through chemical/electrochemical doping enables a theoretical capacity of 295 mAh·g^−1^ [32], positioning it as a competitive cathode candidate for high-performance lithium batteries. The stability of PANI is closely related to its various redox states, such as the fully reduced state, partially oxidized state, and fully oxidized state. Its electrochemical stability is influenced by its molecular structure and doping state, in detail, which can be significantly improved by doping with acids, carbon materials, metal oxides, etc. The electrochemical window under acidic conditions is approximately 1.0 V vs. SCE [33,34,35,36]. Additionally, compared to materials containing heavy metals, non-toxic PANI lowers environmental concerns during manufacture and disposal. The reliance on fossil fuels can be reduced by using sustainable biomass, such as lignin degradation products, as the raw material for PANI (aniline monomer). PANI is a viable option for green cathode materials due to its simple synthesis method, potential environmental friendliness, and capacity for pyrolytic recovery or chemical degradation.

**Figure 2:**
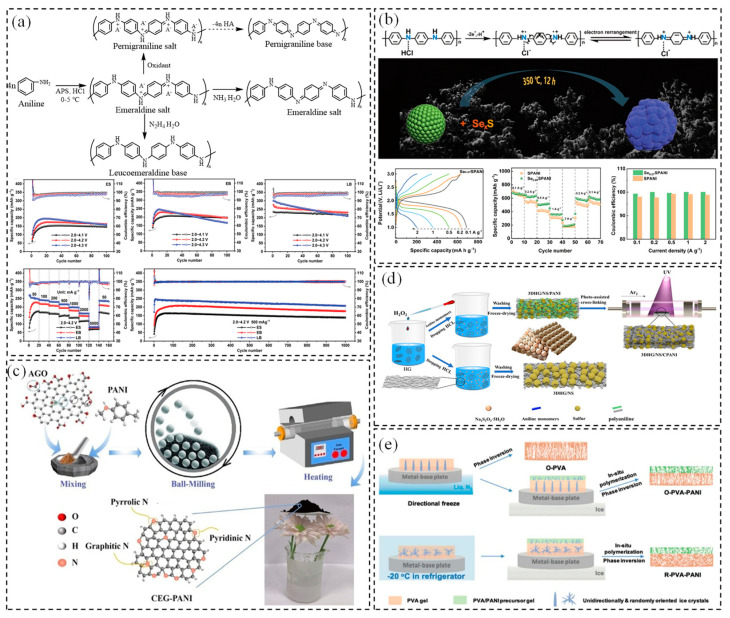
(**a**) Synthesis routes of ES, EB, LB, and their electrochemical performance in 1 M LiClO4/EC–DEC with 1 wt.% FEC [37]; copyright 2023, John Wiley and Sons. (**b**) The generation of quinoid imines, rate performance, coulombic efficiency, and GCD profiles of Se0.07SPANI compared with SPANI cathodes [38]; copyright 2023, Elsevier. (**c**) Synthesis process of CEG–PANI [42]; copyright 2023, Elsevier. (**d**) Preparation diagram of 3DHG/NS/CPANI composite [43]; copyright 2022, Elsevier. (**e**) Schematic illustration of fabrication processes of the oriented neat PVA separator and PVA–PANI Janus separators with oriented pores and random pores [45]. Copyright 2021, Royal Society of Chemistry.

As illustrated in Figure 2a, Guo et al. conducted a comprehensive investigation into the structure–performance relationship of various PANI forms, including emeraldine salt (ES) and emeraldine base (EB), with particular emphasis on the previously underexplored leucoemeraldine base (LB). Through quantitative structural analysis, they confirmed that LB exists in a fully reduced state. The researchers systematically re-evaluated the electrochemical performance of LB in non-aqueous lithium-ion batteries, achieving a remarkable reversible capacity of 197.2 mAh·g^−1^ through optimization of the cycling potential window (1.5–4.4 V) [37]. The optimized LB sample exhibited exceptional electrochemical properties, including a high average coulombic efficiency of 98%, superior rate performance (73.5 mAh·g^−1^ at 1800 mA·g^−1^), and excellent cycling stability with 76% capacity retention after 100 cycles at 20 mA·g^−1^. This groundbreaking study not only deepens the fundamental understanding of PANI’s electrochemical properties and mechanisms but also represents a significant advancement in its development as a cost-effective, high-performance, and environmentally friendly cathode material for energy storage applications, marking a new milestone in this field. Furthermore, various doping strategies have been extensively explored to enhance the electrochemical performance of PANI as a cathode material in lithium batteries. A notable example is the work by Zou et al. [38], who developed a composite positive electrode comprising protonated polyaniline sulfide (SPANI) incorporated into a carbon black and polyvinylidene fluoride matrix (Figure 2b). Electrochemical analysis revealed that the reversible transition between protonated quinone imines (-NH^+^=) and deprotonated quinone imines (-N=) during the charge–discharge process plays a crucial role in promoting lithium polysulfide (LiPS) adsorption and dissociation. This mechanism effectively suppresses the shuttle effect while simultaneously enhancing cycle stability and overall battery performance [39]. Additionally, the electron energy level of the quinone imide structure undergoes optimization through protonation-deprotonation state changes, leading to significantly improved electron conductivity. These investigations demonstrated that protonated PANI sulfide exhibits exceptional electrochemical properties as a cathode-active material [40,41], characterized by superior rate capability, a high reversible specific capacity of 680 mAh·g^−1^ at 0.1 A·g^−1^, and sustained coulombic efficiency approaching 100% during prolonged cycling.

As illustrated in Figure 2c,d, Fu et al. [42] developed an innovative solvent-free synthesis method for preparing graphene nanonetwork–PANI nanocomposites, which demonstrated superior electrical conductivity and enhanced lithium-ion storage capabilities. Acidic graphene oxide and PANI were ball-milled and treated at low temperatures to obtain chemically expanded graphene (CEG-PANI). This method provided sufficient active sites for lithium-ion storage because of the large specific surface area, mesoporous structure, and doping of the PANI conductive agent. In addition, PANI containing N heteroatoms significantly improved the electrochemical properties of CEG-PANI, and increased the reversible capacity to 664 mAh·g^−1^ in 150 cycles at 200 mA·g^−1^ and 253 mAh·g^−1^ in 350 cycles at 2000 mA·g^−1^, respectively. Li et al. [43] implemented a wet etching strategy to introduce the modified PANI to construct a 3D holey graphene (HG)/nano-sulfur (NS)/photo-irradiated polyaniline (CPANI) composite as the cathode material. When PANI was integrated into the graphene structure, its amino groups bonded covalently with epoxy groups or formed hydrogen bonds with hydroxyl groups on the surface of HG. This interaction strengthened the connection between polysulfides and non-polar carbon, enhancing interface contact and improving the structural stability of PANI [44]. This configuration led to a high utilization rate of NS active materials and improved the electrochemical performance of lithium–sulfur batteries.

As illustrated in Figure 2e, Xu et al. prepared a polymer unilateral conductive Janus separator featuring preferentially oriented pores using a water-soluble precursor solution of polyvinyl alcohol (PVA) and PANI through directional freezing and phase conversion [45]. The conductive PANI surface effectively alleviated the issue of localized current concentration and promoted the uniform and stable formation of the solid electrolyte interphase (SEI) layer, which effectively reduced local current density and minimized nucleation sites for lithium dendrite formation. Moreover, the Janus separator enhanced ionic conductivity while maintaining a balance between flexibility and rigidity, alongside superior wrinkle resistance and thermal stability. These contributed to the stable cycling performance and high coulombic efficiency of lithium metal batteries.

### 2.2. Polythiophene (PTH)—PEDOT:PSS

Compared to PANI, PTH exhibits superior flexibility and ductility, making it a preferred choice for conductive polymer composites in flexible electronic applications. Additionally, due to the delocalization of π electrons within its conjugated structure across the entire molecular chain, PTH possesses notable electrical conductivity, excellent stability, and favorable machinability [46]. Furthermore, PTH-PEDOT with a simple molecular structure, small energy gap, and higher conductivity is obtained by reducing the coupling effects of α-β and β-β during the oxidative polymerization process [47,48]. However, PTH-PEDOT is difficult to process and apply because of its poor solubility. To overcome this limitation, PEDOT:PSS is developed by blending PEDOT with the water-soluble polymer polystyrene sulfonic acid (PSS). This composite is widely utilized in energy storage and conversion devices due to its exceptional electron conductivity, high mechanical strength, excellent visible light transmittance, and superior stability [49]. It is worth mentioning that the conductivity of PEDOT:PSS is usually in the range of 10–1000 S cm^−1^, depending on the preparation process and doping degree. By adding some organic solvents (such as DMSO) or using heat treatment, the electrical conductivity can be significantly improved to 10~100 S·cm^−1^. Notably, advanced techniques such as multiple solvent displacement, interface engineering, or nanocomposite formation can enhance PEDOT:PSS conductivity to levels comparable with metals. PEDOT:PSS with doping of PSS exhibits excellent electrochemical stability and can remain stable in various electrolytes even after multiple cycles. Under pH = 7 conditions, its electrochemical window ranges from −1.0 V to 1.0 V vs. Ag/AgCl. Additionally, it remains stable at high potentials, but prolonged exposure to strong oxidizing environments can lead to performance degradation due to the loss of sulfonic acid groups [50,51]. Additionally, the preparation process of PEDOT:PSS typically occurs at low temperatures and pressures, resulting in lower energy consumption. If the use of organic solvents can be minimized, or water-based synthesis techniques are employed, they would be more environmentally friendly. Furthermore, recycling technologies for PEDOT:PSS are being developed to enhance its sustainability [52].

**Figure 3:**
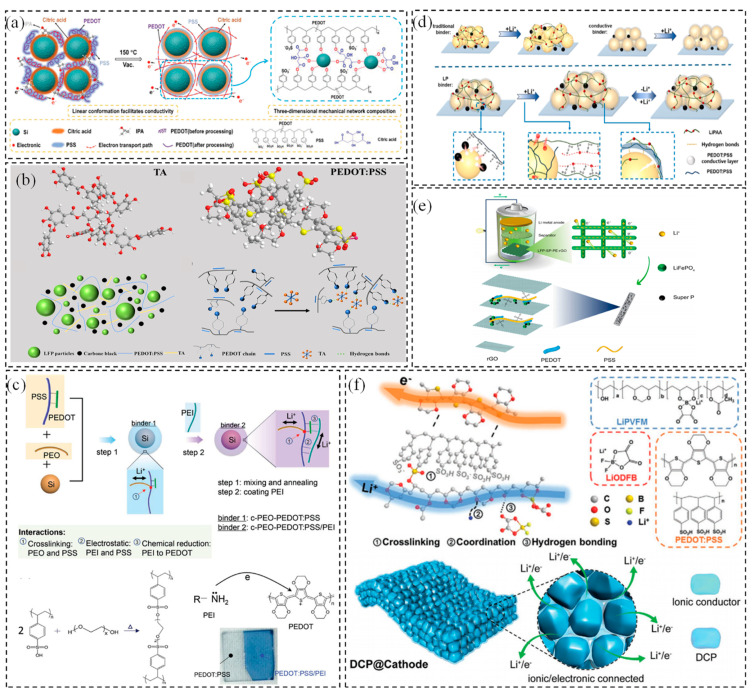
(**a**) Diagram of the preparation of a silicon–based anode electrode material Si/PP/CA from PEDOT:PSS, citric acid, isopropanol, and silicon nanoparticles [54]; copyright 2024, John Wiley and Sons. (**b**) Schematic diagram of PEDOT:PSS composited with TA [55]; copyright 2024, Elsevier. (**c**) PEO and PEI were assembled onto PEDOT:PSS to prepare polymeric adhesives with ionic and electronic conduction [57]; copyright 2018, John Wiley and Sons. (**d**) Schematic illustration of the function mechanism of the LP binder in a Si electrode [58]; copyright 2024, Elsevier. (**e**) Schematic of ions and electrons migration in the LFP–SP–PE–rGO electrodes/separator/Li metal battery system [59]; copyright 2024, Elsevier. (**f**) Sketch of interaction in electronic/ionic dual–conductive polymer between LiPVFM, LiODFB, and PEDOT:PSS, and illustration of Li^+^/electron transportation of DCP–based cathode [60]. Copyright 2020, John Wiley and Sons.

PEDOT:PSS is primarily utilized as a conductive agent and binder in lithium batteries. Zhang et al. [53] developed a standalone PEDOT-coated rhomboid sulfur/single-wall carbon nanotube (SWCNT) flexible composite cathode specifically for lithium–sulfur batteries. The rhomboid sulfur was encapsulated by PEDOT, which served as a shell to effectively capture polysulfides and limit their dissolution during charge/discharge cycles. Simultaneously, it facilitated efficient electron transport and enhanced the flexibility of the electrode. Chen et al. [54] designed a silicon-based negative electrode composite Si-PP-CA composed of PEDOT:PSS, citric acid, isopropanol, and silicon nanoparticles (Figure 3a). As a binder, linear PEDOT improves electron transport efficiency. Meanwhile, the hydroxyl groups in PSS can facilitate the rapid diffusion of Li^+^ and form hydrogen and chemical bonds with citric acid and isopropyl alcohol at the interface, showing a tight interface effect and promoting stable electrochemical performance. In addition, the PEDOT:PSS covered Si particles entirely due to its water-soluble property, which increased the Si content in the electrode. The composite electrode delivered more than 2200 mAh·g^−1^ capacity after 200 cycles at 0.2 A·g^−1^ and retained 89% even at a current density of 1.0 A·g^−1^ after 2000 cycles. As shown in Figure 3b, Li et al. [55] developed a water-soluble TA-PEDOT:PSS (TAPE) binder by mixing PEDOT:PSS with varying ratios of tannic acid (TA) to enhance the stability of LiFePO₄ cathodes in lithium-ion batteries. The sulfonic acid groups in PEDOT:PSS formed hydrogen bonds with the abundant hydroxyl groups in TA [56], altering the linear structure of PEDOT:PSS and establishing π-π stacking interactions between the conjugated π electrons in PEDOT and the aromatic rings in TA. Consequently, the PEDOT:PSS-TA composite exhibited excellent electrical conductivity, mechanical properties, adhesion, and stable electrochemical performance.

In view of the adhesive effect, composite conductive multifunctional polymers were focused on by compositing PEDOT with other materials, for example, PAA, PEO, and graphene, as described in Figure 3c–e. Zeng et al. [57] developed a polymer binder with high ionic and electronic conductivity by integrating the ionic polymers polyethylene oxide (PEO) and polyethylene imine (PEI) into the conductive polymer PEDOT:PSS chain, which yielded a high modulus that helped to maintain the integrity of the Si anode. The lithium-ion diffusivity and electron conductivity of the developed polymer binder were found to be 14 and 90 times higher, respectively, compared to the widely used carboxymethyl cellulose (with acetylene black) binder. Geng et al. [58] introduced a multifunctional binder, LP19, by combining lithiated polyacrylic acid (LiPAA) with PEDOT:PSS. This binder exhibited high electronic and Li-ion conductivity, as well as improved mechanical properties. On a macroscopic scale, PEDOT:PSS facilitated electron transfer between conductive particles and active materials. On a microscopic scale, it established molecular-level electron coupling at the interface between the binder and active particles. This dual mechanism significantly enhanced the rate performance and energy density of the anode. Gao et al. [59] employed 3D printing technology to fabricate a composite electrode with a graded porous multidimensional conductive network. This network consisted of lithium iron phosphate, PEDOT:PSS, and graphene oxide (GO), where PEDOT:PSS and GO served dual roles as adhesive and conductive agents, respectively. The hierarchical porous structure allowed for efficient electrolyte infiltration, while the multidimensional conductive network enhanced electron and ion transport, optimizing the overall performance of the electrode [61,62]. Additionally, PEDOT:PSS and GO were strongly bonded through C-O-S linkages, which boosted mechanical strength and electron transfer efficiency, thereby significantly improving specific capacity and cycle performance. Furthermore, the PEDOT:PSS-based conductive multidimensional network mitigated polarization issues due to the close contact driven by surface negative groups and the enhanced electrical conductivity [63].

In previous work, our team developed an electronic/ionic dual-conductive polymer (DCP) [60] for the composite cathode in solid-state batteries. This polymer was synthesized through intermolecular interactions involving a lithiated polyvinyl formal-derived Li⁺ single-ion conductor (LiPVFM), lithium difluoro borate (LiODFB), and PEDOT:PSS (Figure 3f). The sulfonic acid groups in PEDOT:PSS formed hydrogen bonds with the oxalate groups of LiODFB and the polar groups in LiPVFM, such as carbonyl and amide groups. Crosslinking, coordination, and hydrogen-bonding interactions endowed the DCP with excellent electronic conductivity (68.9 S·cm^−1^), Li-ion conductivity (2.76 × 10^−^⁴ S·cm^−1^), a broad electrochemical window exceeding 6 V, and a high modulus of 6.8 GPa. The DCP enabled the fabrication of self-standing composite cathodes with spatially and temporally stable interfaces, structural integrity, and efficient electronic/ionic transport. These properties enable solid-state batteries to achieve exceptional cycling performance, even under conditions of high active material loading and content.

### 2.3. PBI

PBI is a heterocyclic polymer characterized by a benzimidazole moiety as its repeating unit [64]. It possesses a rigid, rod-like structure with closely packed chain segments, stabilized by strong hydrogen bonding and π-π interactions between the polymer chains. These interactions endow PBI with excellent chemical and thermal stability [65,66]. The imidazole ring in PBI contributes to its electrochemical stability, enabling it to perform well in high-temperature and strongly acidic environments. Covalent organic frameworks (COFs) are materials linked by strong covalent bonds and composed of functional organic structural units [67,68]. PBI can be classified as a unique category of COF materials due to its two-dimensional structure, nitrogen-atom doping, physicochemical stability, and programmable pore size. However, PBI lacks mobile electrons or holes as charge carriers, and its electrical conductivity is below 10^−10^ S·cm^−1^ at room temperature. To enhance its electronic transport capability, PBI can be doped with conductive fillers such as carbon nanotubes, graphene, and carbon black, or modified by introducing conductive units. As a result of these properties, PBI and its derivatives exhibit excellent high-temperature resistance, flame retardance, chemical stability, and mechanical strength, making them promising candidates for electrode materials in lithium batteries [69,70,71]. From an environmental sustainability perspective, the high durability of PBI can extend the lifespan of devices, thereby reducing resource consumption.

**Figure 4:**
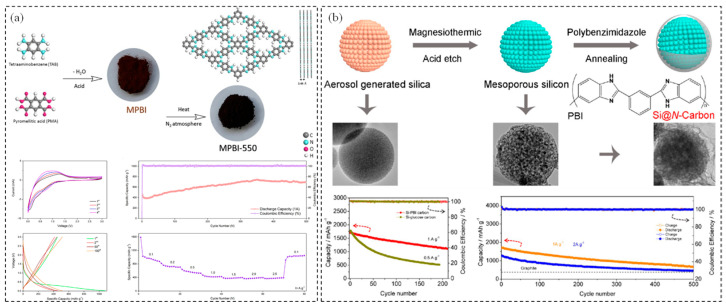
(**a**) Schematic diagram of the synthesis of 2D microporous polybenzimidazole MPBI and its electrochemical results [72]. Copyright 2021, Elsevier. (**b**) Mesoporous silicon anodes synthesized by using polybenzimidazole–derived pyrrolic N-enriched carbon [73]. Copyright 2017, American Chemical Society.

As shown in Figure 4a, Ren et al. [72] synthesized a microporous polybenzimidazole (MPBI) through a condensation reaction between 1,2,4,5-tetraaminobenzene and pyromellitic acid in a polyphosphate medium. Following treatment at 55 °C, MPBI formed a 2D graphene-like framework structure, which maintained a large surface area and preserved nitrogen-atom doping. This structure contributed to its excellent performance, including a remarkable long cycling life, a high reversible capacity of 700 mAh·g^−1^ (at 1 A·g^−1^) after 500 cycles, and outstanding rate performance. Nie et al. [73] developed a pyrrole nitrogen-rich carbon source derived from PBI using an aerosol-assisted assembly and physical adsorption process. As an encapsulating coating for micro-sized silicon spheres, the carbon layers effectively prevented direct contact between the silicon and the electrolyte. This significantly suppressed the formation of an uncontrolled solid electrolyte interphase (SEI) film, thereby enhancing the stability and performance of the silicon-based anode. Furthermore, PBI contained a large number of nitrogen functional groups, which introduced numerous external defects and active sites for lithium storage, improving the specific capacity of the silicon anode [74]. As a result, the mesoporous Si-PBI carbon composite exhibited a high reversible specific capacity (2172 mAh·g^−1^), excellent rate capability (1186 mAh·g^−1^ at 5 A·g^−1^), and a long cycle life, as illustrated in Figure 4b.

### 2.4. PPy

The conductive PPy with a conjugated chain oxidation and corresponding anion doping structure exhibits conductivity ranging from 10^2^ to 10^3^ S·cm^−1^ and demonstrates excellent electrochemical redox reversibility [75]. PPy shows good stability within a moderate potential range (0.76 V vs. Ag/Ag⁺), but it is susceptible to over-oxidation at higher potentials, which can lead to degradation. Studies have shown that the electrochemical stability of PPy can be enhanced by compositing it with carbon materials, metal oxides, and other conductive polymers. Notably, PPy can be produced industrially and applied across various fields due to its numerous advantages, including straightforward preparation methods, non-toxicity, excellent chemical stability, superior mechanical properties, and high conductivity [76,77]. Furthermore, it can be synthesized using electrochemical methods, which minimize the use of excessive chemical reagents and solvents, making it an eco-friendly material.

**Figure 5:**
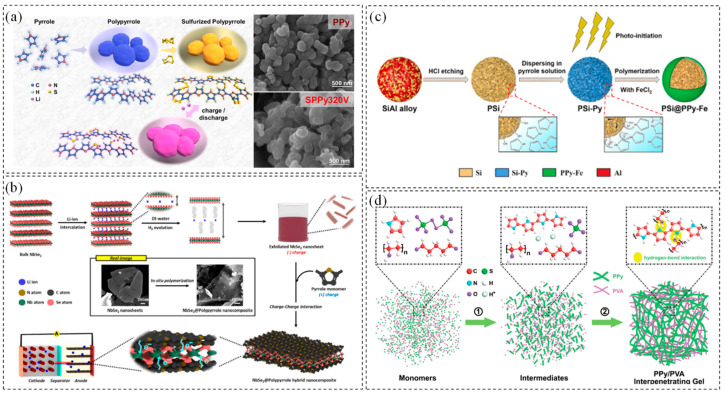
(**a**) Synthesis and discharging process of sulfurized polypyrrole [80]; copyright 2023, Elsevier. (**b**) Schematic illustrations of NbSe_2_/PPy hybrid nanocomposite preparation [81]; copyright 2023, Royal Society of Chemistry. (**c**) Synthetic illustration for PSi/PPy–Fe composites [82]; copyright 2023, Elsevier. (**d**) Structural diagram of the PPy/PVA interpenetrating gel [83]. Copyright 2021, Royal Society of Chemistry.

Due to its unique structure, PPy exhibits high electronic conductivity and mechanical strength [78,79]. Yi et al. [80] synthesized a sulfide polypyrrole (S-PPy) composite at a slightly elevated vulcanization temperature for application in solid-phase converted lithium–sulfur batteries. Unlike conventional mixtures of sulfur and polypyrrole, short-chain sulfur was incorporated into the PPy backbone to form the S-PPy composite. In this structure, PPy provides excellent electronic contact for the short-chain sulfur, enhancing its activity and promoting its conversion into Li_2_S. Additionally, the PPy backbone mitigates the volume changes of sulfur during electrochemical reactions, ensuring the mechanical stability of the electrode and extending its cycling life, as illustrated in Figure 5a.

Byung-Ho Kang et al. [81] demonstrated that NbSe₂, with its metallic properties, can be applied as an anode material by doping it with conductive polypyrrole (PPy), as illustrated in Figure 5b. The π-conjugated structure of PPy was bound to the two-dimensional surface layer of NbSe₂ through van der Waals interactions. Simultaneously, nitrogen-containing functional groups in PPy (such as -NH or -N= on the pyrrole ring) chemically adsorbed onto the surface of unsaturated metal atoms (Nb) or selenium atoms (Se) of NbSe₂. This interaction created a well-dispersed PPy layer on the NbSe₂ surface, enhancing the stability of the composite material. The NbSe₂-PPy hybrid nanocomposites exhibited a lithium storage capacity of 955 mAh·g^−1^, maintained over 300 cycles without capacity loss, and demonstrated excellent rate performance at 4 A·g^−1^. Leveraging the photoelectric effect of semiconductor silicon, Xu et al. [82] developed an eco-friendly and straightforward photoinitiation synthesis approach to fabricate yolk–shell-structured polypyrrole–iron-coated porous silicon microspheres (PSi-PPy-Fe) for use as anode materials in lithium-ion batteries (Figure 5c). The polymerization of pyrrole monomers occurred exclusively at the interface of the silicon matrix, where vacancies were present, effectively preventing the formation of free PPy-Fe particles. Both hydrogen-bond interactions and covalent linkages were established between the PPy-Fe layer and silicon, playing a crucial role in maintaining strong contact and enhancing structural stability [84]. The PPy-Fe coating facilitated faster charge transfer and prevented the silicon-based material from detaching from the copper collector. More significantly, it effectively mitigated the severe structural expansion of the negative silicon electrode.

Additionally, Xia et al. [83] integrated an adhesive polymer into a conductive functional polymer network by mixing polyvinyl alcohol (PVA) and polypyrrole (PPy) to form an interpenetrating gel, as shown in Figure 5d. PPy formed numerous hydrogen bonds with the hydroxyl groups in PVA, enabling its uniform dispersion within the PVA matrix. This prevented agglomeration in the composite material and enhanced both the overall electrical conductivity and mechanical properties. Furthermore, glutaraldehyde promoted cross-linking with PPy at both intramolecular and intermolecular levels, creating stronger bonding effects. This method effectively addressed the brittleness of the electronic conductive polymer and its weak bonding force with the active material, thereby ensuring long-term stability and extending the cycling life of the electrode [85].

This section focuses on the application of typical electrically conductive functional polymers with conjugated structures, such as PANI, PEDOT:PSS, PBI, and PPy in lithium batteries. These conjugated polymers, renowned for their exceptional electrical conductivity, have been extensively utilized as active electrode materials, conductive additives, and/or binders in various battery systems.

PANI is a representative conjugated polymer, distinguished by its high electrical conductivity, excellent electrochemical stability, and environmental durability. Through careful structural and compositional design, PANI-based electrodes demonstrate significantly improved lithium-ion storage performance and electrochemical stability. For instance, PANI-based composites combined with graphene provide a larger specific surface area and a porous structure, which facilitate rapid lithium-ion transport. Additionally, the high conductivity of graphene enhances the charge transfer process. However, the volume expansion of PANI during charging and discharging may compromise the structural integrity of the electrode.

PEDOT:PSS-based multifunctional conductive polymers have been combined with other materials (e.g., tannic acid (TA), polyethylene oxide (PEO), graphene) to improve lithium-ion diffusion and electronic conductivity, thereby enhancing the mechanical integrity and cycling life of the electrode. A key challenge lies in balancing high conductivity with flexibility and stability during prolonged cycling. Through molecular structure design, nanocomposites, and cross-linked networks, it is possible to achieve an optimal balance between these properties. PPy combined with materials like polyvinyl alcohol (PVA) to form interpenetrating network gels demonstrates improved dispersion and conductivity. Additionally, intermolecular crosslinking enhances bond strength, addressing the issues of fragility in conductive polymers and weak binding forces with active materials, such as silicon negative electrode powders. However, PPy faces similar challenges to PANI, including volume expansion and degradation, which can lead to a decline in mechanical properties over prolonged cycling. PBI, as a special class of covalent organic framework (COF) materials, exhibits excellent high-temperature resistance, flame retardance, chemical stability, and mechanical properties, making it a promising candidate for lithium battery electrodes. Strategies such as material selection and composite formulation can mitigate degradation issues during prolonged cycling and high-temperature conditions, thereby enhancing battery safety and performance stability.

In addition to the typical polymers discussed above, numerous other electrically conductive functional polymers (ECFPs) remain to be explored. For instance, polyaniline diamine (PPD) exhibits a high energy storage capacity and holds potential as an electrode material for lithium batteries. However, its poor mechanical properties make it difficult to withstand stress variations during prolonged cycling. Moreover, poly[(2-ethylhexyl) phenylene vinylene)] (MEH-PPV), poly[2,5-bis(3-hexylthiophene-2-yl) thiophene] (PBTTT), poly (triaryl amine) (PTAA), and poly(benzimidazobenzophenanthroline) (BBL) are conjugated polymers known for their excellent optical properties and are widely utilized in organic electronic devices. Because of their poor electrochemical stability under high-voltage operating conditions, the application of these materials in lithium batteries is currently limited. In the future, their mechanical properties could be improved through chemical cross-linking or by compounding with other materials. Additionally, their electrochemical stability may be enhanced by introducing stable side groups, such as fluorinated groups on the polymer backbone, or by doping with inorganic materials like lithium salts or other metal salts. With further optimization through molecular design, modification, and composite formation, a broader range of conjugated polymers is expected to meet the demands for high energy density and extended cycling life in lithium batteries.

## 3. Radical Polymers

Radical polymers are composed of flexible, non-conjugated backbones and functional side groups containing free radicals. These radical sites possess highly localized electrons, distinguishing their structure from that of conjugated conductive polymers. Electron transport in radical polymers is facilitated by the redox reaction processes of the free radicals [86]. This section provides a brief overview of recent advancements in the applications of organic radical polymers, including nitroxide radicals, phenoxy radicals, and others [87,88]. A notable nitroxide free radical species, 2,2,6,6-tetramethylpiperidinyl-n-oxyl (TEMPO), was first reported by Nakahara and his team in 2002 [89]. TEMPO exhibits excellent electrochemical properties, outstanding stability, and rapid redox reactions. Currently, poly (2,2,6,6-tetramethylpiperidinyl methyl acrylate) (PTMA) and poly(norbornene), both of which incorporate TEMPO, have been extensively studied and applied as polymer cathode materials [90,91]. These materials are characterized by low preparation costs, good hydrophilicity, and outstanding electrical conductivity.

### 3.1. Nitroxide Radicals

Nitroxide radicals are named for containing a nitroxide radical motif (N-O·). TEMPO-based materials exhibit intrinsic bipolar characteristics, making them particularly suitable for use as cathode materials due to their high redox potential, typically around 3.6 V vs. Li/Li^+^. The electrochemical stability of nitroxide radicals primarily arises from the nitrogen–oxygen bond (N-O·) within the molecule and the effect of intramolecular electron delocalization. Their electrochemical stability and conductivity can be further enhanced by introducing π-conjugated structures (such as aromatic rings) into the nitroxide radicals to modify their molecular structure or combining them with conductive materials like MXene and porous carbon [92,93,94]. Additionally, nitroxide radicals can be produced from renewable biomass-derived materials. This process enables controlled polymerization with reduced byproducts and waste.

#### 3.1.1. PTMA

The synthesis of PTMA begins with the radical polymerization of the precursor monomer, 2,2,6,6-tetramethylpiperidinyl methyl acrylate (TMPMA), followed by a chemical oxidation step that converts the piperidine ring into a stable nitrogen–oxygen radical. This process endows PTMA with excellent chemical stability and unique electronic properties, making it suitable as a positive electrode material in applications requiring high-rate performance, such as small energy storage devices and fast-charging batteries [95,96]. When used alongside conventional LiFePO₄ or NCM cathodes, PTMA can serve as an additive to optimize conductive networks, thereby enhancing electrochemical performance and prolonging cycling life. Furthermore, PTMA delivers a high output voltage of approximately 3.5 V, which is comparable to that of traditional cathode materials like LiFePO₄. It also offers additional capacity and specific energy density, particularly without the expansion and contraction issues associated with conventional inorganic cathode materials during lithiation and delithiation [97,98].

**Figure 6:**
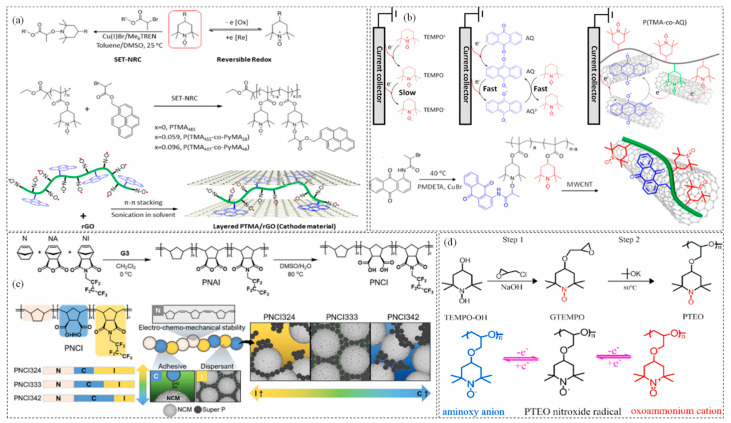
(**a**) Preparation process of PTMA/rGO composites [99]; copyright 2017, American Chemical Society. (**b**) P(TMA–co–AQ) and copolymer/multi–wall carbon nanotube composite electrode [100]; copyright 2022, American Chemical Society. (**c**) Synthesis of PNCI terpolymers and the functional contributions of the PNCI binder [101]; copyright 2023, John Wiley and Sons. (**d**) Synthetic route of PTEO [102]. Copyright 2020, Elsevier.

Zhang et al. developed a layered composite material composed of PTMA and reduced graphene oxide (rGO) through non-covalent π-π stacking, leveraging the advantages of PTMA and the exceptional conductivity and structural stability of rGO. This innovative approach significantly enhanced the cycling performance of lithium batteries at high rates [99]. As illustrated in Figure 6a, PTMA served as the polymer matrix, and the introduction of pyrene groups via chemical modification strengthened its interaction with rGO. This led to the formation of a stable and ordered layered structure, which improved ion transport and cycling stability. Additionally, the nitrogen oxide radicals present in the PTMA chains contributed to the energy storage capacity of the batteries. Li et al. reported the preparation of a composite material, P(TMA-co-AQ), by grafting anthraquinone (AQ) units onto TEMPO polymer chains through a polymerization reaction [100]. The modification of AQ occurred when the quinone groups (C=O) on the chain segments received electrons from reductants, forming a semiquinone radical intermediate (AQ^−^). This intermediate then acted as an electron donor, transferring electrons to TEMPO molecules to accelerate their reduction. As a result, the radical electron structure in TEMPO can stably and persistently accept or transfer electrons, making it less prone to degradation. The P(TMA-co-AQ) composite with multi-walled carbon nanotubes (MWCNTs) as the electrode material exhibited rapid and reversible redox cycling. This grafting structure not only enhanced the stability of TEMPO but also facilitated electron transfer between P(TMA-co-AQ) and the carbon nanotubes. In half-cell tests, AQ-functionalized PTMA demonstrated an initial capacity of 174 mAh·g^−1^ with a capacity loss of 0.18% per cycle, while the discharge capacity based on the TEMPO pair was approximately 85 mAh·g^−1^ (Figure 6b).

#### 3.1.2. Polynorbornene (PNB)

PNB, as a binder, represents a promising approach to enhancing the adhesion and dispersion of electrode components. It is utilized to strengthen the bonding between active material particles. Additionally, PNB’s unique molecular structure provides exceptional mechanical support and chemical stability for the electrodes, alongside its electronic conductivity.

Figure 6c illustrates that Daun Jeong et al. [101] designed a poly (norbornene-co-norbornene dicarboxylic acid-co-heptafluorobutyl norbornene imide) (PNCI)-based binder for nickel-rich layered oxide LiNi_x_Co_y_Mn_z_O_2_ (NCM, x > 0.8) electrodes. Norbornene served as the backbone of the main chain, providing rigidity and mechanical strength, while its fluorinated structure imparted strong hydrophobicity, low surface energy, and high chemical stability. Additionally, the molar composition of the PNCI binder was systematically adjusted to optimize structural integrity, minimizing material loss during charging and discharging. Kan Hatakeyama-Sato et al. [103] synthesized a novel free radical polymer with PNB as the backbone and 2,2,5,5-tetramethyl-1-pyrrolidinyl-N-oxyl (PROXYL) as the side group of unsaturated derivatives. This composite polymer combined a rigid backbone chain with stable nitroxide radical side groups, resulting in a high modulus and good flexibility. These properties enabled the polymer to maintain structural integrity during electrode expansion or contraction. Furthermore, the composite polymer exhibited stable redox capacity as a cathode material, owing to the reversible conversion of the nitroxide radical structure of PROXYL to its reduced state (imine, N-OH).

#### 3.1.3. Poly(tempo-ether-oxetane) (PTEO)

PTEO is a novel organic radical polymer used as a composite electrode material, primarily prepared by incorporating TEMPO with flexible ether chains or oxetane. The nitroxide radicals in PTEO exhibit high efficiency in electron transfer reactions, providing excellent redox activity and high reversibility. The introduction of oxetane or ether groups into the polymer enhances its flexibility and stress resistance, improving the electrode’s adaptability. During the charging process, the nitroxide radical is oxidized, losing electrons to form the oxidation state (N⁺=O). Conversely, during discharging, the nitroxide group is reduced to imine (N-OH) by capturing electrons and lithium ions. Deng et al. incorporated a silica skeleton into the PTEO backbone to construct a highly rigid 3D network structure [102]. As an organic positive electrode, this material can withstand volumetric stresses under high pressure and repeated electrochemical changes. The nitrogen and oxygen radicals in PTEO serve as active centers for energy storage, enabling efficient redox reactions (Figure 6d). The modified PTEO exhibited an electrical conductivity of 0.13 S·m^−1^, surpassing that of other organic cathode materials used in lithium-ion batteries. In performance tests, the PTEO-based lithium-ion battery achieved a capacity of up to 220 mAh·g^−1^ at 0.2 °C, while maintaining capacities of 165 mAh·g^−1^ at 1 °C and 95 mAh·g^−1^ at 8 °C.

### 3.2. Phenoxyl Radicals

Phenoxyl radicals are characterized by extensive conjugated aromatic structures, and their highly efficient reversible transformation between neutral phenoxyl radicals (R-O·) and phenoxyl anions (R-O^−^) plays a crucial role in the energy storage processes of rechargeable batteries. The electrochemical stability of phenoxyl radicals is influenced by molecular structure, electrochemical environment, and substituent effects. For instance, the phenoxenium cation of α-tocopherol exhibits enhanced stability due to intramolecular hydrogen bonding and electron delocalization. Through rational molecular design and optimization of electrochemical conditions, the electrochemical stability of phenoxyl radicals can be significantly improved.

The preparation of phenoxy free radicals involves two main steps: free radical polymerization and chemical oxidation. Specifically, hydrogen galvinoxyl styrene is used as a monomer, which is initiated to generate free radicals at a specific temperature. These free radicals then react with styrene groups to form either dendritic or linear polymers. Subsequently, the hydroxyl group in the hydrogen galvinoxyl group is oxidized to form a free radical (R-O) through chemical oxidation [104,105]. From a green development perspective, phenoxyl radicals can be derived from natural phenolic compounds, such as those extracted from lignin or other plant secondary metabolites. The preparation process can incorporate green chemistry methods, such as aqueous phase systems or photocatalytic processes, to reduce energy and solvent consumption, thereby minimizing environmental pollution.

N-type polymers are polymers that gain electrons during a redox reaction (undergo reduction) and typically function as electron acceptors. These polymers generally exhibit low LUMO (Lowest Unoccupied Molecular Orbital) energy levels, making them suitable candidates for anode materials in batteries. Common examples of n-type polymers include those based on naphthalene diimide (NDI), perylene diimide (PDI), and fullerene derivatives [106]. In contrast, p-type polymers are polymers that lose electrons during a redox reaction (undergo oxidation). These polymers typically possess high HOMO (Highest Occupied Molecular Orbital) energy levels and are commonly used as electron donors and cathode materials in batteries. Common examples of p-type polymers include those based on thiophene (P3HT), benzodithiophene (BDT), and carbazole [107].

**Figure 7:**
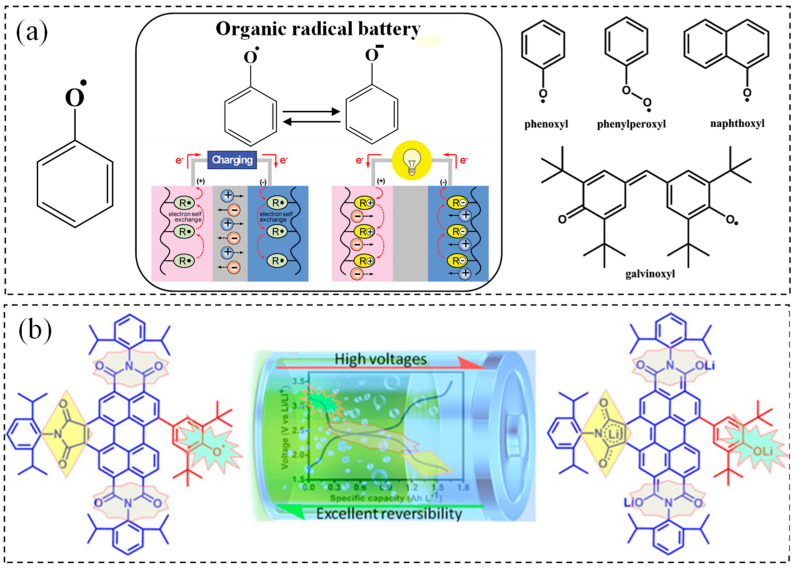
(**a**) The derivatives and applications of phenoxy radicals [109]; copyright 2009, John Wiley and Sons. (**b**) Stable radical preparation process by C–C coupling of phenoxy radical with PDI or BPTIs [110]. Copyright 2018, John Wiley and Sons.

As depicted in Figure 7a,b, Takeo Suga et al. investigated the reversible and rapid one-electron redox reaction between phenoxyl radicals and phenoxyl anions [108]. They explored an n-type and redox-active radical polymer with galvinoxyl as the anode active material. Building on this, a totally organic radical battery was developed with the n-type redox-active radical polymer as the anode and a p-type radical polymer as the cathode [109]. Li et al. [110] reported the synthesis of two stable bifunctional radicals prepared through C-C coupling of redox-active phenoxyl radicals with perylene diimides (PDIs) or benzo perylene triimides (BPTIs). Incorporating electron-deficient PDIs or BPTIs into phenoxyl radicals increased the density of redox-active groups per molecule, elevated the redox potential, and enhanced the stability of the phenoxyl radicals.

### 3.3. Hydrazyl Radicals

Hydrazyl radicals are a class of organic free radicals centered around nitrogen, characterized by the presence of hydrazine groups (-N•-N=). These radicals are typically generated through the oxidation or dehydrogenation of hydrazine compounds (R-NH-NR’). The general formula for hydrazyl radicals is R-N•-NR′ or R₂N-N•-R, where at least one nitrogen atom carries an unpaired electron. The electrochemical stability of hydrazyl radicals can be significantly enhanced by introducing specific electron-withdrawing groups or by designing particular molecular structures, such as bulky substituents or conjugated systems. Experimentally, their electrochemical stability is typically characterized using cyclic voltammetry (CV) and electron paramagnetic resonance (EPR) [111]. Hydrazyl radicals are generally synthesized from hydrazine and aromatic compounds, involving organic hydrazine derivatives that usually rely on petrochemical resources. Recently, the synthesis process has been optimized to reduce byproducts or select alternative reagents. Hydrazyl radicals exhibit high reactivity, which can lower catalyst requirements and improve chemical conversion efficiency, highlighting their potential as green chemistry candidates.

**Figure 8:**
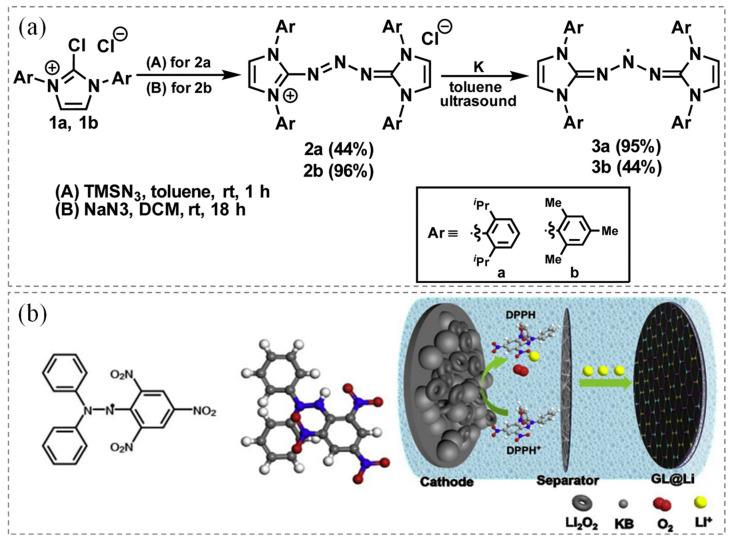
(**a**) Synthesis of N–heterocyclic carbenes supported triazenyl radicals [112]. Copyright 2017, American Chemical Society. (**b**) Molecular structure of DPPH and its charging reaction mechanism in Li–O_2_ batteries [114]. Copyright 2020, Elsevier.

Lee et al. [112] reported the synthesis and characterization of stable triazenyl radicals, which were obtained through the single-electron reduction of the corresponding triazenyl cations using potassium metal (Figure 8a). Among these, the triazine radical with a hydrazine structure can be oxidized to form a triazine cation. When treated with a transition metal source or an electrophilic reagent, these radicals reversibly oxidize back into cations while extracting a hydrogen atom from heteranthrene, forming an N-H bond at the central nitrogen atom [113]. Additionally, due to their aromaticity and conjugated systems, triazenyl radicals exhibit high chemical stability and can undergo multiple redox reactions. Furthermore, lightweight triazenyl radicals delivered a theoretical specific capacity of up to 200 mAh·g^−1^ as positive active materials in lithium-ion batteries. Triazenyl radicals can also serve as redox media to promote the oxidation of Li_2_O_2_ particles, reducing the overpotential in lithium–oxygen batteries. In Figure 8b, Bai et al. [114] designed a redox medium utilizing the nitrogen-containing free radical 1,1-diphenyl-2-pyridine hydrazyl radical 2,2-diphenyl-1-(2,4,6-trinitrobenzene) hydrazyl (DPPH). DPPH significantly enhanced the solubility and migration rate of oxygen, improved charge transport, and facilitated the decomposition of Li_2_O_2_. Consequently, lithium–oxygen batteries incorporating DPPH as an intermediate molecule achieved a low terminal charge voltage of 4.12 V and maintained stable performance over 80 cycles at a high current density of 400 mA·g^−1^ with a fixed capacity of 1000 mAh·g^−1^.

In this part, the application and progress of several radical polymers, including nitroxide free radicals, phenoxy free radicals, and hydrazine free radicals, in lithium batteries are reviewed. Their synthesis methods, electrochemical properties, and strategies for functional improvement are discussed in detail. These polymers are composed of flexible scaffolds and side groups bearing free radicals with highly localized electrons, enabling them to exhibit excellent electron transport kinetics and electrochemical performance.

PTMA is a typical nitroxide radical polymer known for its excellent chemical stability and electron transport capabilities, making it a suitable candidate for use as an electrode material. However, its higher molecular weight and fewer active sites limit its stability in higher voltage ranges. Another nitroxide radical polymer, PNB, exhibits adhesive properties and can serve as a binder for electrode materials. It is crucial to note that the incorporation level of PNB significantly influences the electrochemical performance of the electrodes. Therefore, its large-scale application still depends on further research, including the optimization of adhesion behavior in solutions and improvement of compatibility with existing electrode systems. Composite electrodes combining NCM with PNB demonstrate outstanding mechanical properties, chemical stability, and conductivity, enhancing energy density and cycling stability. Furthermore, phenoxy radicals prepared through free radical polymerization and chemical oxidation possess efficient reversible electron transfer capabilities. Research indicates that binding with electron-deficient molecules, such as perylene diimides or benzo perylene triimides, is one of the most effective strategies for improving the cycling life of batteries based on phenoxy free radicals.

Additionally, some free radical polymers, such as hydrocarbon radicals and halogen radicals, have been reported to exhibit high redox reaction activity. However, when utilized as electrode active materials in lithium batteries, hydrocarbon radicals can induce the decomposition of organic electrolytes, significantly compromising the cycling lifespan and safety of the batteries. Although this issue can be mitigated through the design of radical scavengers or reactive additives, it remains a considerable challenge. In contrast, halogen radicals readily react with organic electrolytes and electrode materials, leading to unstable side reactions. To address this, research has focused on chemical modification or doping to adjust the reactivity of halogen radicals, thereby reducing their adverse impact on the electrochemical properties of batteries.

## 4. Conclusions and Outlook

ECFPs have emerged as a promising class of materials for advanced energy storage applications, combining electron transport capabilities, lithium storage reactivity, structural flexibility, and molecular design versatility. This review focuses on two major categories of ECFPs: conjugated polymers and free radical polymers.

For conjugated polymers, the molecular design and structural optimization of the π-conjugated skeleton can significantly enhance electron transport capacity and electrochemical performance. Specific strategies include main-chain extension, side-chain modification, orientation control, doping, and composite formation. Main-chain extension aims to reduce the π-π stacking distance and lower the energy band gap, which decreases the resistance to electron and hole mobility, thereby improving conductivity. Orientation control involves aligning polymer segments using external forces, promoting the orderly arrangement of molecular chains and enhancing charge carrier mobility. Side-chain engineering often incorporates functional groups, such as electron donors (D) and electron acceptors (A), to improve charge separation and transport characteristics. Additionally, doping with protonic acids, oxidants, ionic liquids, or carbon nanotubes can significantly enhance charge transport efficiency. Composite formation with materials such as graphene or carbon nanotubes is a widely accepted strategy to improve electrical conductivity, mechanical properties, and electrode stability. The applications of conjugated polymers in energy storage systems have garnered significant attention. For instance, PANI- and PPy-based materials demonstrate exceptional polysulfide capture ability and high-rate performance in lithium–sulfur batteries.

On the other hand, radical polymers achieve enhanced stability and electrochemical performance through rational molecular design. This includes the strategic incorporation of stable radical moieties (e.g., hydroxylamine radicals, enoxy radicals, and nitrogen–sulfur radicals), main/side-chain engineering to optimize radical density and electronic structure, and flexible chain integration (e.g., ether/oxetane segments in PTEO) for improved volume adaptability. By compositing with conductive materials such as reduced graphite oxide or multi-walled carbon nanotubes, free radical polymers can build conductive networks, significantly enhancing electron transport capacity. Additionally, the development of multifunctional binder systems, such as adhesive PNB derivatives, demonstrates dual benefits: enhanced interparticle adhesion in composite electrodes and improved mechanical strength and chemical stability.

In the future, the development of ECFPs will focus on three critical areas: synthetic innovation, multifunctional integration, and system optimization. Synthetic innovation will prioritize eco-friendly and scalable synthesis methodologies. For example, the development of water-based polymer systems or biocatalytic synthesis pathways for the preparation of PANI aims to replace traditional toxic solvents, such as NMP. Similarly, enzyme-catalyzed polymerization or photoinitiated polymerization for PPY preparation can reduce residues of oxidants like FeCl_3_. Multifunctional integration will involve designing adaptive architectures for diverse energy storage platforms. PANI/PEDOT:PSS heterojunctions can be developed by utilizing the pseudo-capacitance of PANI alongside the high conductivity of PEDOT:PSS. Furthermore, the synergy between redox activity and conductivity allows for the incorporation of nitroxide radicals onto the PPY backbone. The optimization of the system will focus on balancing energy density, rate capability, and cycling durability through hierarchical structural design, interface engineering, and the development of advanced binders. For instance, nanotube arrays combined with microporous carbon can optimize the ionic/electronic transport pathways of PANI. Gradient-doped multilayer films can effectively balance the surface conductivity of PEDOT:PSS with adhesion to underlying layers. Porous frameworks loaded with radical molecules can enhance the utilization of active sites in radical materials. Furthermore, temperature-sensitive or light-responsive adhesives based on PBI or PEDOT:PSS can enable the dynamic regulation of electrode structures. The continued evolution of ECFPs in molecular design, performance optimization, and sustainable manufacturing positions these materials as pivotal components in next-generation energy storage and conversion technology, offering wide-ranging prospects for their applications in energy storage and conversion devices.

## Data Availability

No new data were created or analyzed in this study.

## Abbreviations

The following abbreviations are used in this manuscript:

ECFPs	Electrically conductive functional polymers
PANI	Polyanilines
PEDOT	Poly(3,4-ethylenedioxythiophene)
PSS	Polystyrene sulfonic acid
PBI	Polybenzimidazole
PPy	Polypyrroles
PVA	Polyvinyl alcohol
PTH	Polythiophene
TA	Tannic acid
PEO	Polyethylene oxide
PEI	Polyethylene imine
COF	Covalent organic skeletons
TEMPO	2,2,6,6-tetramethylpiperidinyl-n-oxyl
PTEO	Poly (tempo-ether-oxetane)
AQ	Anthraquinone
PNB	Polynorbornene
PROXYL	2,2,5,5-tetramethyl-1-pyrrolidinyl-N-oxyl
PPD	polyaniline diamine
MEH-PPV	Poly[(2-ethylhexyl) phenylene vinylene)]
PBTTT	Poly[2,5-bis(3-hexylthiophene-2-yl) thiophene]
PTAA	Poly(triaryl amine)
BBL	Poly(benzimidazobenzophenanthroline)
